# Adaptive Role of Inversion Polymorphism of *Drosophila subobscura* in Lead Stressed Environment

**DOI:** 10.1371/journal.pone.0131270

**Published:** 2015-06-23

**Authors:** Bojan Kenig, Zorana Kurbalija Novičić, Aleksandra Patenković, Marina Stamenković-Radak, Marko Anđelković

**Affiliations:** 1 Department of Genetics of Populations and Ecogenotoxicology, Institute for Biological Research “Siniša Stanković”, University of Belgrade, Belgrade, Serbia; 2 Chair of Genetics and Evolution, Faculty of Biology, University of Belgrade, Belgrade, Serbia; 3 Serbian Academy of Sciences and Arts, Belgrade, Serbia; CSIRO, AUSTRALIA

## Abstract

Local adaptation to environmental stress at different levels of genetic polymorphism in various plants and animals has been documented through evolution of heavy metal tolerance. We used samples of *Drosophila subobscura* populations from two differently polluted environments to analyze the change of chromosomal inversion polymorphism as genetic marker during laboratory exposure to lead. Exposure to environmental contamination can affect the genetic content within a particular inversion and produce targets for selection in populations from different environments. The aims were to discover whether the inversion polymorphism is shaped by the local natural environments, and if lead as a selection pressure would cause adaptive divergence of two populations during the multigenerational laboratory experiment. The results showed that populations retain signatures from past contamination events, and that heavy metal pollution can cause adaptive changes in population. Differences in inversion polymorphism between the two populations increased over generations under lead contamination in the laboratory. The inversion polymorphism of population originating from the more polluted natural environment was more stable during the experiment, both under conditions with and without lead. Therefore, results showed that inversion polymorphism as a genetic marker reflects a strong signature of adaptation to the local environment, and that historical demographic events and selection are important for both prediction of evolutionary potential and long-term viability of natural populations.

## Introduction

Anthropogenic pollutants in the environment, including heavy metals, affect natural populations of organisms in many ways. One of the most significant outcomes with potentially large long-term effects is genetic change. Exposure to pollutants can cause selection pressure which may lead to genetic adaptation [1, 2]. Evidence for local adaptation to environmental stress on various levels of genetic polymorphism of different species has been provided in numerous empirical works, with documented evolution of heavy metal tolerance [3–5].

Chromosomal inversion polymorphism is known to be associated with environmental changes and local adaptation [6–12]. Several hypotheses have been suggested to explain the maintenance of inversion polymorphism in natural populations (reviewed in Hoffmann and Rieseberg [10]). The traditional explanation for the spread and evolution of the inversions by Dobzhansky [6] is that alleles within inversions have epistatic effects on fitness, and that combinations of the alleles are “coadapted” by having higher fitness than predicted from the sum of their independent effects. An alternative hypothesis states that inversions are favored because they bring together locally adapted alleles even without epistasis [13]. Both of these hypotheses explain that the reduced recombination between inversion heterokaryotypes avoids gene exchange with other genetic backgrounds. The suppression of recombination in heterokaryotypes within or close to inverted regions may facilitate the capture of locally adaptive alleles across multiple linked loci, thus allowing inversions to spread through a local population [6, 11, 13].

*Drosophila subobscura* Collin (Diptera: Drosophilidae) possesses one of the richest inversion polymorphism in the genus, on all five acrocentric chromosomes with more than 60 different inversions, forming more than 90 different chromosomal gene arrangements described. Some of the inversions are rare and/or restricted to limited areas [7]. Inversion polymorphism in *D*. *subobscura* is to a certain degree temporally and spatially associated with the variation and dynamics of ecological factors, and it shows clear-cut geographic, habitat-associated, and seasonal variations [9, 14–17]. Therefore, chromosomal inversions of *D*. *subobscura* are adaptive in response to environmental changes and represent a suitable model for studying adaptation to a changing environment. Moreover, *Drosophila* studies allow for detailed multigenerational genetic analyses in precisely controlled conditions [18], which facilitates better assessment of the effects under long-term chronic changes [19, 20]. The effect of history of heavy metal pollution on population fitness response to laboratory lead contamination in *D*. *subobscura* showed that a higher resistance was present in a population originating from the more polluted locality [21]. It was also found that the extent of inversion polymorphism was higher in urban *Drosophila* populations, which was a result of the large number of ecological niches in this type of environment [22, 23]. However, only few studies have analyzed how historical differences in inversion polymorphisms affect the ability of populations to respond to new environmental conditions, although the prediction of population-specific genetic response to heavy metal pollution is one of the most important tasks of ecotoxicology [24, 25].

The present study analyzes the dynamics and variation of inversion polymorphisms in *D*. *subobscura* flies originating from two natural populations, sampled from ecologically distinct habitats. The two populations were maintained in laboratory conditions with different levels of lead concentrations during six generations. The aims were:

1. to analyze initial population differentiation regarding the inversion frequencies, and monitor whether such differentiation would be retained over generations in the experimental conditions or whether the selection pressure imposed by lead would cause a convergence of populations to a similar level of inversion polymorphism.
2. to detect the level of inversion polymorphism within and between populations maintained on different lead concentration across generations.
3. to check if heterozygosity (percent of heterokaryotypes) would remain the same over generations or it would change (increase or decrease) under the lead pollution, and furthermore, whether this change would differ between populations.

## Materials and Methods

Two populations of *Drosophila subobscura* were sampled, one at the locality of Deliblato Sands (DS) (*Orno-Quercetum cerris-virgiliane*), about 60 km northeast from Belgrade, Serbia (44° 49' N; 21° 07' E), and the other in the Botanical Garden (BG) (*Arboretum*), in an urban part of Belgrade, capital of Serbia (44° 49' N; 20° 28' E). Verbal permissions for collecting the flies were obtained for both localities. For the Botanical garden, permission was obtained from the management of the Botanical Garden "Jevremovac", The University of Belgrade, and at the locality Deliblato Sands from the Forest Office “Deliblato”. The species studied is not classified as endangered or protected, the localities are not on protected land, and therefore no additional specific permissions were required for collection.

The literature data for lead concentration in soil sampled in DS (5–35 mg/kg) characterize this particular locality as unpolluted [26]. The BG locality has chronically been exposed to anthropogenic activity, mostly related to traffic pollution. The soil in this area is heavily polluted, with the average concentration of lead in the soil of 298.6 mg/kg [27, 28].

The flies were collected by using fermented fruit traps. Approximately 100 isofemale lines (IF) per population were made, each from a single gravid wild-caught female. All lines were maintained and all experiments performed under constant laboratory conditions: temperature of 19°C, approx. 60% relative humidity, light of 300 lux and 12/12 h light/dark cycles. After two generations, three pairs of males and females from each IF line were used to establish two synthetic mass populations in order to preserve the original genetic variability. The F_1_ progeny of the mass populations was used to establish three experimental groups with three different media. The control group was reared on the standard *Drosophila* medium (water/cornmeal/yeast/sugar/agar/nipagine), without lead. In order to determine the toxic potential of lead acetate, eight different concentrations of lead acetate were prepared in triplicate for treating *D*. *subobscura* eggs. Exactly 100 eggs per concentration were used and the number of emerged adults was recorded. Based on the results obtained, a survival curve was made, and two concentrations of lead acetate [Pb (CH_3_COO)_2_·3H_2_O] were chosen. The first experimental group (low lead concentration group–LLC) had 10 μg/mL of lead acetate added to the medium, which is a sublethal concentration. The second experimental group (high lead concentration group–HLC) had 100 μg/mL of lead acetate added to the medium, which represented 20% of the lethal concentration (LC _20_). Flies in all three groups (control, LLC, HLC) were kept *en masse*, each group consisting of 10 bottles (replicates) containing 25 mL of the medium. The flies from the F_1_ generation were mixed randomly within each group, and the F_2_ generation was initiated with 50 adult flies in an equal sex ratio for each bottle (500 adults in total per experimental group). Adult females were allowed to lay eggs for the first seven days after they were placed into the bottles. After that period, the adults were removed, and newly emerged adult flies were used to initiate the next generation. Each subsequent generation was obtained exactly in the same manner.

It was shown by Kenig et al [21] that these two populations initially showed different resistance to lead in laboratory conditions. Egg to adult viability was higher in the Botanical Garden population compared to Deliblato Sands population, and the difference was more expressed on media with both low and high concentrations of lead than on the control medium. Furthermore, the viability increased across generations in the HLC group from DS, while in the corresponding group from BG there were no significant changes across generations (viability remained high in all generations). Therefore, the initial higher resistance to contamination, meaning higher fecundity, higher viability, and faster egg to adult development in all the generations studied, was found in *D*. *subobscura* originating from the locality of Botanical Garden.

An analysis of inversion polymorphism was carried out with males from DS and BG populations in F_1_, F_3_, and F_6_ generations, from each experimental group (control, LLC and HLC). The males were individually crossed with virgin females from the Küsnacht laboratory line, which is homokaryotypic for all acrocentric chromosomes of the set (A_ST_, J_ST_, U_ST_, E_ST_, and O_ST_). Salivary glands from third-instar larvae were squashed and their chromosomes stained with aceto-orcein solution. In order to minimize the possibility of error in determining the karyotype and to reduce the probability of wrong determination to (1/2)^8^ for each chromosome, we analyzed eight larvae from the progeny of each cross. For the cytological analysis of chromosome arrangements, the chromosome map of Kunze-Mühl and Müller [29] was used, as well as nomenclature according to Kunze-Mühl and Sperlich [30].

About 30 males (240 autosomes and 30 sex chromosomes) from each experimental group (C, LLC, HLC) in three generations (F_1_, F_3_ and F_6_), in both populations (DS and BG) were analyzed, making a total of about 420 males.

A G-test was done to determine the homogeneity of chromosome arrangement frequencies within all five chromosomes, in pairwise comparisons among experimental groups (C, LLC and HLC), within generations (F_1_, F_3_ and F_6_), for each population (DS and BG). Z-statistics was used to assess the differences in frequencies of individual chromosome arrangements. Both tests were performed in pairwise comparisons among experimental groups and among generations, for each population (intra-population differences), and between the populations (inter-population differences). The inversion polymorphism parameters—the degree of heterozygosity (HZ) and Index of free recombination (IFR) were derived from arrangement frequencies according to the description of Krimbas [7]. The IFR takes in consideration the length of the chromosome region within inversions, but there is considerable evidence that inversions affect recombination beyond their breakpoints [31]. Therefore, HZ may be a preferable metric for the degree of inversion polymorphism. A sequential Bonferroni test [32] was used to adjust for the multiple pairwise comparisons within each experimental group.

## Results

### Intra-population differences in parameters of chromosomal polymorphism

The chromosome arrangement frequencies, heterozygosity, and the index of free recombination (IFR) were calculated for *D*. *subobscura* from Deliblato Sands (Table 1) and Botanical Garden (Table 2) populations, in three generations (F_1_, F_3_ and F_6_) on standard medium (control group) and media with two lead concentrations (LLC and HLC). The inversion polymorphism parameters (heterozygosity and IFR) were similar in all experimental groups and generations, except in the controls from F_3_ and F_6_, which showed a lower value of IFR and an increase in heterozygosity compared to the F_1_ generation. Trends of variation of these parameters were found to be similar in both populations (Tables 1 and 2).

**Table 1:** Frequencies of chromosomal arrangements in *D*. *subobscura* population from Deliblato Sands (DS), in F3 and F6 generation in the control group and in the groups on low (LLC group—10 μg/mL) and high (HLC group—100 μg/mL) concentrations of lead.

	DS (F1)	F3 generation			F6 generation		
Chromosomal arrangements	(n = 66)	control (n = 64)	LLC (n = 64)	HLC (n = 66)	control (n = 64)	LLC (n = 60)	HLC (n = 64)
Ast	0,545	0,625	0,406	0,364	0,375	0,567	0,500
A1	0,455	0,344	0,531	0,636	0,531	0,433	0,500
A2	-	0,031	0,063	-	0,094	-	-
Jst	0,242	0,391	0,281	0,197	0,266	0,150	0,234
J1	0,758	0,609	0,719	0,803	0,734	0,850	0,766
Ust	0,121	0,219	0,188	0,288	0,109	0,150	0,141
U1+2	0,712	0,672	0,641	0,606	0,672	0,733	0,797
U1+2+6	0,167	0,109	0,172	0,106	0,219	0,117	0,063
Est	0,288	0,375	0,500	0,424	0,281	0,183	0,313
E8	0,530	0,438	0,297	0,394	0,344	0,567	0,672
E1+2+9	0,182	0,156	0,188	0,167	0,328	0,233	0,016
E1+2+9+12	-	0,031	0,016	0,015	0,047	0,017	-
Ost	0,364	0,391	0,422	0,424	0,344	0,500	0,578
O6	-	-	-	-	-	-	-
O3+4	0,379	0,266	0,406	0,318	0,516	0,400	0,344
O3+4+1	0,197	0,344	0,172	0,212	0,109	0,083	0,063
O3+4+2	0,061	-	-	0,045	0,031	0,017	0,016
HZ	0,485	0,570	0,508	0,523	0,578	0,425	0,422
IFR	85,074	78,889	82,434	81,327	80,355	85,153	86,403

n = number of chromosomes analyzed; HZ = degree of heterozygosity; IFR = Index of free recombination.

**Table 2:** Frequencies of chromosomal arrangements in *D*. *subobscura* population from the Botanical Garden (BG), in F3 and F6 generations in the control group and in the groups on low (LLC group—10 μg/mL) and high (HLC group—100 μg/mL) concentrations of lead.

	BG (F1)	F3 generation			F6 generation		
Chromosomal arrangements	(n = 62)	control (n = 60)	LLC (n = 64)	HLC (n = 64)	control (n = 64)	LLC (n = 64)	HLC (n = 64)
Ast	0,516	0,633	0,594	0,813	0,531	0,594	0,688
A1	0,419	0,367	0,406	0,125	0,438	0,375	0,313
A2	0,065	-	-	0,063	0,031	0,031	0,000
Jst	0,419	0,367	0,266	0,313	0,328	0,281	0,344
J1	0,581	0,633	0,734	0,688	0,672	0,719	0,656
Ust	0,145	0,233	0,156	0,156	0,281	0,188	0,172
U1+2	0,677	0,617	0,734	0,719	0,609	0,719	0,672
U1+2+6	0,177	0,150	0,109	0,125	0,109	0,094	0,156
Est	0,355	0,400	0,547	0,500	0,484	0,547	0,719
E8	0,371	0,383	0,219	0,328	0,219	0,203	0,156
E1+2+9	0,258	0,183	0,219	0,156	0,234	0,234	0,125
E1+2+9+12	0,016	0,033	0,016	0,016	0,063	0,016	-
Ost	0,468	0,400	0,328	0,500	0,406	0,484	0,531
O6	-	-	-	-	0,016	-	-
O3+4	0,403	0,450	0,453	0,375	0,359	0,438	0,281
O3+4+1	0,113	0,083	0,141	0,078	0,156	0,078	0,156
O3+4+2	0,016	0,067	0,078	0,047	0,063	-	0,031
HZ	0,589	0,675	0,500	0,617	0,703	0,555	0,477
IFR	81,581	76,806	82,339	80,191	75,373	81,556	83,998

n = number of chromosomes analyzed; HZ = degree of heterozygosity; IFR = Index of free recombination.

The statistical significance for differences in chromosomal arrangement frequencies between generations (F_1_, F_3_ and F_6_) within experimental groups (C, LLC and HLC) for both populations (DS and BG) is given in Table 3. Significant differences between generations were obtained for the DS population in all experimental groups. In the control group, differences were found between F_3_ and F_6_ for complex gene arrangements E_1+2+9_ (p < 0.05), O_3+4_ (p < 0.001) and O_3+4+1_ (p < 0.001), and between F_1_ and F_6_ generations for E_8_ inversion (p < 0.05). In the LLC group, significant differences between F_3_ and F_6_ generations were found only in frequencies of two E chromosome arrangements: E_St_ (p < 0.001) and E_8_ (p < 0.001). The highest intergenerational difference between F_3_ and F_6_ was found for HLC groups, in frequencies of arrangements on chromosomes U (U_St_: p < 0.05; U_1+2_: p < 0.01), E (E_8_: p < 0.001; E_1+2+9_: p < 0.001) and the O_3+4_ arrangement (p < 0.01) (Table 3).

**Table 3:** Differences in the frequencies of individual chromosomal arrangements of *D. subobscura* between generations (F1, F3, F6) in the experimental groups (C, LLC, HLC) from Deliblato Sands (DS) and from the Botanical Garden (BG).

exp.group	C						LLC		HLC	
generation	F1/F3		F3/F6		F1/F6		F3/F6		F3/F6	
population	DS	BG	DS	BG	DS	BG	DS	BG	DS	BG
Ast										
A1										
A2										
Jst										
J1										
Ust									2.888 *	
U1+2									- 3.357 **	
U1+2+6										
Est							5.239 ***			- 3.587 **
E8						3.031 *	- 4.294 ***		- 4.49 ***	3.210 *
E1+2+9			- 3.21 *						4.207 ***	
E1+2+9+12										
Ost										
O6										
O3+4			- 4.099 ***						3.493 **	
O3+4+1			4.479 ***							
O3+4+2								3.226 *		

p<0.05 *

p<0.01 **

p<0.001 ***

Z-test values are given only for significant comparisons. p-values are corrected for multiple comparisons.

The population from BG showed significant differences only between generations F_3_ and F_6_: within the LLC group for the O_3+4+2_ gene arrangement (p < 0.05), and in the HLC group for two E chromosome arrangements (E_St_: p < 0.01; E_8_: p < 0.05) (Table 3).

Differences in the frequencies of chromosome arrangements among experimental groups (C, LLC, HLC) within generations (F_1_, F_3_, F_6_) from DS and BG are shown in S1 Table. The population from DS showed a significantly lower frequency of the complex chromosome arrangement O_3+4+1_ in the LLC group within generation F_3_ when compared to the control group (p < 0.05). Significant differences were found in F_3_ between the control and the HLC group, for frequencies of chromosome arrangements on chromosome A (A_St_: p < 0.05; A_1_: p < 0.05) and chromosome J (J_St_: p < 0.01; J_1_: p < 0.01).

A slightly different result was obtained within the F_6_ generation. Significant differences were found in frequencies of arrangements on chromosomes U, O, and the most significant on E, among experimental groups. The increase of the E_8_ frequency is positively associated with the lead concentration in the medium, revealed by a significant difference in the frequency of this inversion between the control and both groups with lead (p < 0.01 for LLC; p < 0.001 for HLC). The HLC group had a significantly lower frequency of E_1+2+9_ compared to both the control and the LLC group (p < 0.001). A significant difference in frequency of the U_1+2+6_ was detected between the control and the HLC group (p < 0.01) (S1 Table). Another significant difference in frequencies of the standard gene arrangement on chromosome O was registered between the control and the HLC group (p < 0.01).

When the experimental groups from the BG population within F_3_ and F_6_ generations were compared, a few statistically significant differences were found. Within the F_3_ generation, a significantly lower frequency of A_1_ chromosome inversion in HLC group was detected compared to the control (p < 0.05) and LLC group (p < 0.01). Within the F_6_ generation the only significant increase was obtained in the frequency of the E_ST_ arrangement in the HLC group, compared to the control (p < 0.01) (S1 Table).

In both populations (S2 and S3 Tables) significant differences in chromosome arrangement frequencies (per chromosome) were found between the experimental groups (C, LLC and HLC) within generations (F_3_ and F_6_). The results of the G test for both populations are in accordance with the results of the Z-statistics.

### Inter-population differences in parameters of chromosomal polymorphism

Chromosomal polymorphism analysis of the initial generation (F_1_) showed that the Deliblato Sands population had 14 structural types, while there were 16 in the Botanical Garden population. This evidence pointed to a higher level of chromosomal variability in the BG population, which was indirectly confirmed by differences in IFR values, where BG population had lower IFR (81.581) compared to DS (85.074).

In the control groups of F_3_ generation, significant differences were found in the distribution of chromosome O gene arrangements between the two populations (Table 4). The arrangement O_3+4_ had a higher frequency in the control group from BG (p < 0.05), O_3+4+1_ had higher frequency in DS (p < 0.001), while O_3+4+2_ was completely absent in the control group from DS, but present in BG in low frequency (p < 0.05). Significant differences between arrangements of chromosome O were also confirmed by the G-test (p < 0.001) as shown in S4 Table. In the F_6_ generation the G-test revealed differences between two populations (p < 0.05) in the distribution of gene arrangements on the U chromosome in the control groups. Standard arrangements of chromosomes U and E had lower frequency in population DS, and the Z-test confirmed significance of these interpopulation differences: U_St_ (p < 0.01) and E_St_ (p < 0.05) (Table 4). The heterozygosity on the control in the F_3_ generation was higher (0.675) in the BG population than in DS (0.570). The IFR value in the DS control group was 78.889, which is higher than the one obtained for BG (76.806). The heterozygosity was higher by 12.5% in the BG control group in the F_6_ generation (0.703) than in the corresponding group from DS (0.578). Furthermore, the IFR values were very different, and the value of this parameter in the DS control was 80.355, while in the corresponding group from BG it was 75.373 (Tables 1 and 2).

**Table 4:** Differences in the frequencies of individual chromosomal arrangements of *D*. *subobscura* between populations Deliblato Sands and Botanical Garden (DS/BG comparisons) within experimental groups (C, LLC, HLC) and generations (F3, F6).

generations	F3			F6		
arrangements	C	LLC	HLC	C	LLC	HLC
Ast			5.193 ***			
A1			- 5.992 ***			
A2						
Jst						
J1						
Ust				3.468 **		
U1+2						
U1+2+6						
Est				3.343 *	5.926 ***	6.503 ***
E8					- 5.900 ***	- 8.375 ***
E1+2+9						3.422 **
E1+2+9+12						
Ost						
O6						
O3+4	3.034 *					
O3+4+1	-4.968 ***		- 3.058 *			
O3+4+2	2.971 *	3.226 *				

p<0.05 *

p<0.01 **

p<0.001 ***

Z-test values are given only for significant comparisons. p-values are corrected for multiple comparisons.

Between the LLC groups of DS and BG populations, in the F_3_ generation, there were significant differences only for gene arrangement O_3+4+2_ (p<0.05) due to the absence of this arrangement in the DS group (Table 4). The G-test did not reveal any significant difference between these groups from two populations in F3 for any of the analyzed chromosomes (S4 Table). In the F_6_ generation there were significant differences in frequencies of gene arrangements of chromosome E between two populations on LLC (Table 4). Arrangement E_St_ had a significantly lower frequency in the LLC group from DS than the one from BG (p < 0.001), while E_8_ inversion showed the opposite; the lower frequency was found in the LLC group from BG (p < 0.001). Significant interpopulation differences between the LLC groups for chromosome E (p < 0.001) and for all chromosomes (p < 0.05) were also revealed by the G-test (S4 Table).

The heterozygosity was similar in both populations (DS—0.508 and BG—0.500), as well as the IFR values (DS—82.434 and BG—82.339). In generation F_6_ the heterozygosity was higher in the LLC from population BG (0.555) compared to the corresponding group originating from DS (0.425), which represents 13% of the difference. The IFR value in the group reared on lower lead concentration from DS was 85.153 and from BG was 81 (Tables 1 and 2).

In the F_3_ generation, in the HLC group, the standard gene arrangement of chromosome A showed a significantly higher frequency in BG than the corresponding group from DS (z = 5.193, p < 0.001), while A_1_ inversion showed the opposite (z = -5.992, p < 0.001) (Table 4). Significant difference between these groups was also found for the O_3+4+1_ complex gene arrangement, with a higher frequency in DS than in BG (z = -3.058, p < 0.05). The G-test confirmed significant interpopulation differences in the F_3_ generation among HLC groups, on chromosome A, and among all chromosomes (G = 20.71, p < 0.001; G = 32.00, p < 0.01) (S4 Table). In the F_6_ generation between HLC groups significant differences were found in the frequencies of gene arrangements on chromosome E. Arrangement E_St_ had significantly lower frequency in the HLC group from DS than in the corresponding group from BG (z = 6.503, p < 0.001), while for the E_8_ inversion there was a lower frequency in the HLC group from BG (z = -8.375, p < 0.001). Arrangement E_1+2+9_ showed a higher frequency in the BG group reared on higher lead concentration through six generations (z = 3.422, p < 0.01) (Table 4). The G-test confirmed significant inter-population differences in the distribution of gene arrangements between HLC groups in the F_6_ generation, on chromosome E (G = 38.86, p < 0.001) and among all chromosomes (G = 50.14, p < 0.001) (S4 Table). Heterozygosity was higher in the BG population in both generations: in the F_3_ generation by 9.4%, and in the F_6_ generation by 5.5%. The value of IFR in the HLC group from DS was 86.403, while the corresponding value from BG was 83.998 (Tables 1 and 2).

## Discussion

The general goal of the present study was to determine the importance of *D*. *subobscura* inversion polymorphism in a lead polluted environment, as well as the importance of this type of polymorphism in the ability to adapt to environmental changes. By transferring the individuals originating from the populations with different background of environmental pollution into constant laboratory conditions with and without lead contamination, this study aimed to reveal the role of population history and selection in the evolution of inversion polymorphism.

### Intrapopulation variability (Deliblato Sands)

Laboratory conditions affected the change of gene arrangements frequencies, both between generations and treatments in the Deliblato Sands population. Even on the control medium, significant differences between F_3_ and F_6_ generations and between F_1_ and F_6_ generations in the frequencies of E and O chromosome arrangements were found, which possibly resulted from the population’s adaptation to laboratory conditions. The transfer of individuals from the natural environment leads to a process of a population’s biological and genetic refinement in response to such an environmental change [33, 34]. It has been shown that the response to this type of environmental disruption is population-specific for *D*. *subobscura* [35, 36]**.** Adaptation to the laboratory conditions probably depends on some selective advantages of the chromosomal arrangements carried by individuals, or to their linkage with a gene arrangement already participating in a major heterotic association, as predicted by Carson [37]. If natural populations of *Drosophila* are set up with balanced systems of co-adapted genes in inversions, as proposed by the Dobzhansky’s hypothesis [6], transfer to the laboratory may break down such a genetic balance and enable a new co-adapted system to evolve. This could be supported by the hypothesis of Lewontin [38] stating that genetic polymorphism should be lost in a uniform environment, while it could also be in accordance with the idea of Carson [37] stating that genetic polymorphism may be lost in a uniform environment only if each heterozygote was particularly adapted to some slightly different environmental variables in nature, not present in laboratory conditions.

Significant differences in gene arrangement frequencies between the F_3_ and F_6_ generations were found for arrangements on the E chromosome (E_st_ and E_8_) in the LLC group, and on chromosomes U (U_st_, U_1+2_), E (E_8_, E_1+2+9_), and O (O_3+4_) in the HLC group. Changes in inversion polymorphism parameters which were positively or negatively associated with lead were, among other causes, the result of epistatic or additive effect of the gene content of the inversions. Additionally, when we looked at the frequency of chromosome arrangements within the generations, we observed that the initial response to the presence of lead was associated with chromosomes A and J, while the prolonged period of such conditions included changes in the arrangements frequency of the E chromosome. The changes in the E and O chromosome arrangement frequencies were in accordance with previous studies of *D*. *subobscura* inversion polymorphism dynamics in population cages [33]. It was found that under such conditions, those particular chromosomes acted as flexible systems under predominant diversifying selection. Furthermore, a great divergence of sex chromosome arrangement frequencies was detected on the higher lead concentration. It was shown that increased tolerance to cadmium in *D*. *melanogaster* was linked to the presence of sex chromosome that originated from partially cadmium-resistant line of the species [39], as well as that one or more genes responsible for metallothionein structural gene expression regulation were located on sex chromosome [40]. The A_1_ inversion in the DS population showed a significant change in frequency after three generations of exposure to lead. Although the inversion A_1_ is of a simple type, it covers a considerable region of the chromosome, with a great number of loci carrying different combinations of alleles.

### Intrapopulation variability (Botanical Garden)

The results obtained on inversion polymorphism variability for experimental groups originating from the Botanical Garden showed significant differences in gene arrangement frequencies both between generations and treatments, and no significant difference between generations within the control group. Although arrangement O_3+4+2_ appeared already at a low frequency, it decreased over generations, especially on the LLC. Also, a significant change in gene arrangement frequencies on the E chromosome (E_st_ and E_8_) for the HLC group between generations was obtained, suggesting that genes of the E and O chromosomes have an important role in the response to lead pollution. Studies of heavy metal resistance acquisition in various *D*. *melanogaster* lines have shown that if one regulatory gene is included in the resistance mechanism, a strong pleiotropic effect of that gene leads to changes in fitness components. It is unlikely that more regulatory genes have a strong pleiotropic effect [41]. Genes that are involved in the resistance mechanism exhibit strong epistatic effects on the loci of allelogenic combinations in chromosomal inversion. Epistasis between genes involved in the resistance mechanism and the rest of the genome occurs during acquisition of resistance to heavy metals [10, 42].

### Inter-population variability

Initial inter-population differences in chromosome arrangement frequencies were expected if we take into account the previous analysis of chromosomal polymorphism of the populations from the same locations [23]. The differences are due to the diverse evolutionary history of each population originating from ecologically distinct habitats. Historical population differentiation remained high and even increased during the experiment, particularly in the lead treated groups. Initially, the BG population showed a significantly higher level of chromosomal variability compared to the DS population. It has already been shown that there is a positive correlation between the complexity of urban environment and the degree of inversion polymorphism [22, 43]. The genetic variability in the BG population, affected by a high anthropogenic activity, is most probably shaped by disruptive selection. Intraspecies and intrapopulation competition promotes diversification of the number and variability of ecological niches. In such conditions, different phenotypes occupy different niches, which promote less competition for resource usage. Disruptive selection is seen in high density populations rather than in low density populations, due to the fact that higher density populations often compete more intensely for resources, which either drives polymorphisms or creates changes in niches in order to relax competition [44].

The transfer from nature to standard laboratory conditions caused changes across generations in the frequencies of E and O chromosome arrangements in the DS control group, which indicates a recreation of genetic content inside inversions of these chromosomes. The absence of corresponding changes of the chromosomal arrangement frequency in the BG population control group indicates that sets of alleles within inversions of this population enabled a more efficient response to laboratory conditions [11, 22, 43]. Such a different response is in accordance with the hypothesis that populations of DS and BG have different sets of alleles inside the chromosomal arrangements and different existing adaptive potentials, shaped by the local environmental conditions of their native habitats. These differences remain high even after six generations, which is in accordance with the results of Fragata et al. [45]. They found that a strong initial historical signature is maintained in the pattern of inversion frequencies in different *D*. *subobscura* populations, even after 40 generations of evolution in a common laboratory environment, regardless of the clear pattern of the convergent evolution on the level of live history traits [46].

Detected population differentiation remained high, and even increased, during the experiment in the lead treatment groups. In both DS and BG populations, we found inter-generational changes in gene arrangement frequencies of the E and O chromosomes, related to the presence of lead at both concentrations. Therefore, it is clear that individuals from this population are more sensitive to lead pollution in laboratory conditions, since they were not adapted to this kind of environmental stress, compared to the BG population originating from more polluted environmental background. The genetic structure of each population is shaped by current natural selection, but is also the outcome of the historical processes in a particular population. Demographic factors can affect the genetic content of a given inversion, producing different targets for selection between populations [6, 11, 13]. The structure of gene arrangements, as well as the specific combinations of those arrangements in different populations, determines the way that environmental stress can affect fitness. Differences in structure and integrity of the genetic systems of DS and BG led to a population-specific response. According to the Dobzhansky’s co-adaptation hypothesis [6], it is expected that inversions of geographically distant populations will differ in genetic content. Nevertheless, most studies did not find genetic differentiation within inversions, nor did they suggest low genetic differentiation within inversions across the European cline of *D*. *subobscura*, [47, 48]. In contrast with these results, Santos [49] detected recombination load in crosses of homokaryotypic *D*. *subobscura* lines for several inversions of the O chromosome, which shows that the genetic content of a given inversion can change even inside the same population. Our results do not exclude a local adaptation scenario [13], in which the chromosomal arrangements of a particular population carry combinations of alleles adapted to factors in a given local environment. Positive fitness effect of allelic combination involved in a local adaptation does not necessarily involve epistatic interactions, but the result from a cumulative effect of these combinations [47].

Although heterozygosity was different in initial generations between the populations DS and BG, we recorded similar changes in both populations, namely a negative correlation with the lead concentration. This trend was more expressed in the BG population, and enhanced in the F_6_ generation. However, under laboratory conditions without lead, an increase of heterozygosity in both populations was recorded, especially in BG. We have already mentioned that in *D*. *subobscura*, chromosomal inversions suppress recombination in heterokaryotypes, which may help to maintain positive epistatic interactions among groups of alleles at loci contained in the inversion [7]. Three mutually not exclusive balancing selection mechanisms have been suggested for the maintenance of inversion polymorphism: co-adaptation [50], supergene selection [51], and local adaptation [52]. Genetic exchange among chromosomes in heterokaryotypes from different populations would disrupt locally adapted allele complexes and produce unfit offspring. Consequently, homokaryotypes could suffer an extra disadvantage due to disruption of these favorable combinations, since their chromosomes can freely recombine. Our results suggest that lead contamination affected the composition of the genetic content in both populations through a decrease of heterozygosity and an increase of the portion of chromosomal segments which are ready to be subjected to recombination, a process that could lead to an increase of the recombination load. Although recombination load leads to fitness decrease because the recombination breaks up the associations between beneficial combinations of interacting alleles [11, 13, 52], new allele combinations could potentially have an advantage in novel environmental conditions such as lead contamination.

## Conclusions

Based on the analysis of an adaptive genetic marker, such as chromosomal inversion polymorphism, we showed that populations retain signatures from past contamination events, and that heavy metal pollution can provoke adaptive changes in population. Our results show not only that the initial differences in inversion polymorphisms between populations remained throughout the laboratory experiment, but they even increased in conditions of lead contamination. We also observed that a population originating from a more polluted natural habitat had higher initial level of inversion polymorphism, which was also more stable during the experiment, both in conditions with and without lead. Past processes of environmental contamination can result in the ability for evolutionary change, and thus can lead to adaptive divergence of populations residing in differently polluted habitats. Therefore, we showed that both historical events, as well as selection processes, are important for prediction of evolutionary potential and long-term viability of natural populations, especially for further deliberation in parts of evolutionary ecology and conservation biology.

## Supporting Information

S1 Table Z-test for inversion frequencies between groups. (DOCX)

S2 Table G test for inversion frequencies between groups of DS. (DOCX)

S3 Table G test for inversion frequencies between groups of BG. (DOCX)

S4 Table G test for inversion frequencies between populations. (DOCX)
