# Supplementary material for: Adaptive Role of Inversion Polymorphism of Drosophila subobscura in Lead Stressed Environment
Source: PLoS One. 2015 Jun 23;10(6):e0131270. doi: 10.1371/journal.pone.0131270 (PMC4478027; doi:10.1371/journal.pone.0131270)
Supplement: S1 Table — (DOCX) [file pone.0131270.s001.docx]

**S1 Table. Z-test for inversion frequencies between groups.**

| Differences in frequencies of individual chromosomal arrangements of *D. subobscura* between experimental groups (C, LLC, HLC) within generations (F3, F6) from Deliblato Sands (DS) and from the Botanical Garden (BG). | | | | | | |
| --- | --- | --- | --- | --- | --- | --- |
|  |  |  |  |  |  |  |
| generation | F3 | | | | | |
| exp.group | C/LLC | | LLC/HLC | | C/HLC | |
| population | DS | BG | DS | BG | DS | BG |
|  |  |  |  |  |  |  |
| Ast |  |  |  |  | 2.980 * |  |
| A1 |  |  |  | 3.602 ** | - 3.337 * | 3.142 * |
| A2 |  |  |  |  |  |  |
| Jst |  |  |  |  | 3.433 ** |  |
| J1 |  |  |  |  | - 3.433 ** |  |
| Ust |  |  |  |  |  |  |
| U1+2 |  |  |  |  |  |  |
| U1+2+6 |  |  |  |  |  |  |
| Est |  |  |  |  |  |  |
| E8 |  |  |  |  |  |  |
| E1+2+9 |  |  |  |  |  |  |
| E1+2+9+12 |  |  |  |  |  |  |
| Ost |  |  |  |  |  |  |
| O6 |  |  |  |  |  |  |
| O3+4 |  |  |  |  |  |  |
| O3+4+1 | 3.143 * |  |  |  |  |  |
| O3+4+2 |  |  |  |  |  |  |
|  | | | | | | |
| generation | F6 | | | | | |
| exp.group | C/LLC | | LLC/HLC | | C/HLC | |
| population | DS | BG | DS | BG | DS | BG |
|  |  |  |  |  |  |  |
| Ast |  |  |  |  |  |  |
| A1 |  |  |  |  |  |  |
| A2 |  |  |  |  |  |  |
| Jst |  |  |  |  |  |  |
| J1 |  |  |  |  |  |  |
| Ust |  |  |  |  |  |  |
| U1+2 |  |  |  |  |  |  |
| U1+2+6 |  |  |  |  | 3.596 ** |  |
| Est |  |  |  |  |  | - 3.830 ** |
| E8 | - 3.527 ** |  |  |  | - 5.251 *** |  |
| E1+2+9 |  |  | 5.257 *** |  | 6.627 *** |  |
| E1+2+9+12 |  |  |  |  |  |  |
| Ost |  |  |  |  | - 3.761 ** |  |
| O6 |  |  |  |  |  |  |
| O3+4 |  |  |  |  |  |  |
| O3+4+1 |  |  |  |  |  |  |
| O3+4+2 |  |  |  |  |  |  |
| p<0.05 *, p<0.01 **, p<0.001 ***  Z-test values are given only for significant comparisons.  p-values are corrected for multiple comparisons. | | | | | | |
