# Supplementary material for: Adaptive Role of Inversion Polymorphism of Drosophila subobscura in Lead Stressed Environment
Source: PLoS One. 2015 Jun 23;10(6):e0131270. doi: 10.1371/journal.pone.0131270 (PMC4478027; doi:10.1371/journal.pone.0131270)
Supplement: S2 Table — (DOCX) [file pone.0131270.s002.docx]

| Results of the G test for chromosomal arrangement frequencies in pairwise comparisons among experimental groups (C, LLC, HLC) originating from Deliblato Sands (DS) within generations (F3, F6). | | | | | | |
| --- | --- | --- | --- | --- | --- | --- |
| Generation | F3 | | | F6 | | |
| Chromosomes | C/LLC | C/HLC | LLC/HLC | C/LLC | C/HLC | LLC/HLC |
| A |  | 6.57 * |  |  |  |  |
| J |  | 5.95 * |  |  |  |  |
| U |  |  |  |  | 6.82 * |  |
| E |  |  |  |  | 33.53 *** | 18.41 *** |
| O |  |  |  |  |  |  |
| all |  |  |  |  | 52.52 *** |  |
| p<0.05 *, p<0.01 **, p<0.001 *** | | | | | | |

**S2 Table. G test for inversion frequencies between groups of DS.**
