# Supplementary material for: Adaptive Role of Inversion Polymorphism of Drosophila subobscura in Lead Stressed Environment
Source: PLoS One. 2015 Jun 23;10(6):e0131270. doi: 10.1371/journal.pone.0131270 (PMC4478027; doi:10.1371/journal.pone.0131270)
Supplement: S4 Table — (DOCX) [file pone.0131270.s004.docx]

| Results of G test for chromosomal arrangement frequencies in pairwise comparisons between populations Deliblato Sands and Botanical Garden (DS/BG comparisons) within experimental groups (C, LLC, HLC) and generations (F3, F6). | | | | | | |
| --- | --- | --- | --- | --- | --- | --- |
|  |  |  |  |  |  |  |
|  |  |  |  |  |  |  |
| generations | F3 | | | F6 | | |
| chromosomes | C | LLC | HLC | C | LLC | HLC |
| A |  |  | 20.71 *** |  |  |  |
| J |  |  |  |  |  |  |
| U |  |  |  | 7.58 * |  |  |
| E |  |  |  |  | 22.79 *** | 38.86 *** |
| O | 19.28 *** |  |  |  |  |  |
| all |  |  | 32.00 ** |  | 29.45 * | 50.14 *** |
| p<0.05 *, p<0.01 **, p<0.001 *** | | | | | | |

**S4 Table. G test for inversion frequencies between populations.**
